# Circulating Exosomes from Septic Mice Activate NF-κB/MIR17HG Pathway in Macrophages

**DOI:** 10.3390/biomedicines12030534

**Published:** 2024-02-27

**Authors:** Shao-Chun Wu, Cheng-Shyuan Rau, Yi-Chan Wu, Chia-Jung Wu, Chia-Wen Tsai, Lien-Hung Huang, Chia-Wei Lin, Tsu-Hsiang Lu, Ming-Yu Yang, Ching-Hua Hsieh

**Affiliations:** 1 Department of Anesthesiology, Kaohsiung Chang Gung Memorial Hospital, Chang Gung University College of Medicine, Kaohsiung 83301, Taiwan; shaochunwu@gmail.com; 2 Graduate Institute of Clinical Medical Sciences, Chang Gung University College of Medicine, Taoyuan 33302, Taiwan; yangmy@mail.cgu.edu.tw; 3 Department of Neurosurgery, Kaohsiung Chang Gung Memorial Hospital, Chang Gung University College of Medicine, Kaohsiung 83301, Taiwan; ersh2127@adm.cgmh.org.tw; 4 Department of Plastic Surgery, Kaohsiung Chang Gung Memorial Hospital, Chang Gung University College of Medicine, Kaohsiung 83301, Taiwan; janewu0922@gmail.com (Y.-C.W.); alice8818@yahoo.com.tw (C.-J.W.); flying011401@gmail.com (C.-W.T.); ahonbob@gmail.com (L.-H.H.); sallylin1201@gmail.com (C.-W.L.); rabbit670326@yahoo.com.tw (T.-H.L.)

**Keywords:** sepsis, exosome, nuclear factor-kappa B (NF-κB), long non-coding RNA (lncRNA), miR-17-92a-1 cluster host gene (MIR17HG), pull-down assay

## Abstract

Circulating exosomes derived from polymicrobial sepsis contain various non-coding RNAs and proteins. Isobaric tags for a relative or absolute quantitation proteomic analysis of the exosomal content revealed 70 dysregulated proteins in the circulating exosomes from septic mice. Next-generation sequencing was used to profile the long non-coding RNA expression in primary cultured macrophages treated with exosomes obtained from the blood of septic C57BL/6 mice, and it was discovered that the nuclear factor-kappa B (NF-κB)/miR-17-92a-1 cluster host gene (MIR17HG) pathways were activated in the macrophages. The inhibition of MIR17HG expression by RNA interference resulted in significantly decreased cell viability. RNA pull-down assays of MIR17HG revealed that ten protein targets bind to MIR17HG. Interaction networks of proteins pulled down by MIR17HG were constructed using GeneMANIA, and their functions were mainly involved in ribonucleoprotein granules, type I interferons, the regulation of organelle assembly, the biosynthesis of acetyl coenzyme A, as a signal transducer and activator of transcription (STAT) protein phosphorylation, and mRNA splicing. Furthermore, RNA interference inhibited MIR17HG expression, resulting in significantly decreased cell survival. In conclusion, this work discovered considerable MIR17HG overexpression in macrophages treated with circulating exosomes from sepsis-affected animals. This study’s findings assist us in comprehending the role of exosomes in modulating inflammatory responses and mediating pathogenic pathways in macrophages during sepsis.

## 1. Introduction

Exosomes can be absorbed by target cells through endocytosis and fusion and are involved in intercellular communication [1,2]. They can transport various biological molecules responsible for the signaling pathways between immune cells [3,4,5]. In sepsis, exosomes play a crucial role in regulating inflammatory response [6,7] and mediating pathogenic pathways across tissues [8]. Exosomes are thought to be involved in macrophage activation, thereby causing a corresponding inflammatory response [9,10,11]. The activation of macrophages by sepsis-associated exosomes is considered to contribute significantly to sepsis development by perpetuating the inflammatory cascade and exacerbating tissue damage [2,9,12]. However, the precise mechanisms underlying macrophage activation by circulating exosomes during sepsis remain unclear.

Long non-coding RNAs (lncRNAs) are a class of RNA molecules longer than 200 nucleotides that do not encode proteins [13,14,15]. LncRNAs participate in various mechanisms involved in gene expression at multiple levels by interacting with RNA-binding proteins [16], DNA, and other RNA molecules [17,18]. In sepsis, lncRNAs regulate inflammation by acting as molecular scaffolds or decoys [19,20], influencing the expression of genes involved in inflammation [21,22], and modulating immune cell function and macrophage polarization [23,24]. LncRNAs can have both enhancing and suppressing effects on immune functions during sepsis [25], affecting the balance between pro-inflammatory and anti-inflammatory responses [26,27,28] and ultimately influencing the development of sepsis-associated organ dysfunction [22,29,30,31,32,33,34,35,36,37].

Exosomes have been shown to modulate inflammation in a variety of immune-mediated disorders other than sepsis, including inflammatory bowel disease [38], allergic airway diseases [39], Parkinson’s disease [40], retinal diseases [41], transplantation rejection [42], and cancer [43]. However, the effect of circulating exosomes on macrophages during sepsis remains largely unknown. In this study, next-generation sequencing was used to investigate lncRNA expression in macrophages treated with exosomes obtained from the blood of mice with sepsis and it was discovered that the nuclear factor-kappa B (NF-κB)/miR-17-92a-1 cluster host gene (MIR17HG) pathways were activated in the macrophages. Furthermore, the inhibition of MIR17HG expression significantly decreased cell viability. RNA pull-down assays revealed ten protein targets that bind to MIR17HG, and interaction networks were constructed based on their interconnections to illustrate their functions.

## 2. Materials and Methods

### 2.1. Sepsis Mouse Model of CLP

This study used male C57BL/6 mice weighing 25–30 g, which were obtained from the National Laboratory Animal Center, NARLabs, Taiwan. In addition, The Jackson Laboratory (JAX stock #006100, Bar Harbour, ME, USA) provided transgenic B10.Cg-H2k Tg(NFkB/Fos-luc)26Rinc/J mice that express the luciferase gene triggered by two copies of the NF-B regulatory element in any tissue. To induce a moderate level of sepsis, a surgical procedure was performed by making a midline abdominal incision and ligating the cecum below the ileocecal valve using the standard technique [44]. The CLP animal model of high-grade sepsis involved creating a single puncture in the cecum using a 21-G needle with 75% of the cecum being ligated. Under such condition, 80% of mice died within 1 d and all mice died within 4 d after CLP induction (Supplementary File S3, *n* = 20). A small quantity of feces was extracted from the cecum to induce polymicrobial peritonitis. The abdominal wall was closed in two layers. In the sham operation group, the same procedure was performed, which included opening the peritoneum and exposing the bowel without tying off the cecum or puncturing it with a needle. Following surgery, the mice were revived by subcutaneously injecting 5 mL of pre-warmed normal saline per 100 g body weight. All the animals were housed in a hospital facility accredited by the Association for Assessment and Accreditation of Laboratory Animal Care International. The analgesia and surgical procedures followed both national and institutional regulations. The mice were kept in a room at approximately 25 °C, humidity maintained at 60 ± 10%, and a 12 h light–dark cycle. They had access to pellets and water on demand, ensuring access to food and hydration throughout the study.

### 2.2. Source of Primary Cultured Macrophages

Macrophages were obtained from the femoral and tibial bone marrow of C57BL/6 mice or B10.Cg-H2k Tg(NFkB/Fos-luc)26Rinc/J mice using primary culture techniques. Mice were anesthetized, and the femoral and tibial bones were carefully dissected and placed in sterile culture containers containing PBS. A sterile 25-gauge needle was used to infuse sterile Dulbecco’s Modified Eagle Medium or Roswell Park Memorial Institute (RPMI) 1640 medium into the bones, flushing out the bone marrow. The flushed bone marrow suspension was collected in sterile tubes.

To obtain a single-cell suspension, the collected bone marrow suspension was gently pipetted up and down to break the remaining tissue clumps. Next, ammonium–chloride–potassium lysing buffer was added to the suspension to lyse the red blood cells. The suspension was incubated at 37 °C for 1–5 min to allow for lysis. The lysis buffer was neutralized by adding an equal volume of sterile RPMI 1640 medium containing 10 µg/mL macrophage colony-stimulating factor and 10% fetal bovine serum from Sigma-Aldrich (Sigma-Aldrich, St. Louis, MO, USA.).

To pellet the cells, the suspension was centrifuged at 300× *g* for 5 min at 37 °C. The resulting cell pellet was gently resuspended and transferred onto a sterile cell culture plate. The plate was placed in a humidified incubator set at 37 °C with 5% CO_2_. The cells were maintained by changing the culture medium every 2–3 days. After a culture period of 21 days, the macrophages adhered to the culture plate and were ready for harvesting. A cell dissociation reagent was used to detach the macrophages from the plate.

### 2.3. Purification of the Circulating Exosomes of Mice

In this study, exosomes purified from the blood of mice (*n* = 20) subjected to CLP were isolated and referred to as CLP-exo, whereas exosomes from sham-operated mice (*n* = 20) served as the control exo. After intracardiac puncture, 1 mL of blood was extracted from each mouse to obtain exosomes. Serum was then separated by centrifugation at 3000 rpm for 10 min to remove any precipitates or contaminants.

ExoQuick Exosome Precipitation Solution (System Biosciences, Mountain View, CA, USA) was used to purify exosomes from the serum. To initiate the purification process, 63 μL of ExoQuick precipitation solution was added to 250 μL of the supernatant. The mixture was thoroughly mixed and incubated at 4 °C for 30 min. After incubation, the combined solution was centrifuged at 1500× *g* for 30 min. Finally, the supernatant was removed by aspiration, and the mixture was centrifuged for 5 min to separate any remaining liquid. The pellet containing the exosomes was then resuspended in 100 μL PBS.

### 2.4. Characterization of the Purified Circulating Exosomes

Exosomes were characterized following the guidelines outlined in Minimal Information for Studies of Extracellular Vesicles 2022 [31,32]. Western blotting was performed to evaluate the expression of exosomal surface markers on purified exosomes, using the culture medium as a control. Exosomal proteins were extracted and transferred onto polyvinylidene fluoride membranes by polyacrylamide gel electrophoresis. The membranes were then incubated overnight at 4 °C with primary antibodies against various exosomal markers, including CD9 (catalog number ab92726; Abcam, Cambridge, Cambs, UK), CD81 (catalog number ab109201; Abcam), TSG101 (catalog number ab30871; Abcam), and Flotillin-1 (catalog number ab78178; Abcam). Primary antibodies were diluted to a concentration of 1:500–1:1000 for the incubation. The membranes were then washed three times with a 0.1% TBS/Tween 20 solution at 37 °C. Subsequently, they were incubated for 2 h with a secondary antibody that was conjugated with horseradish peroxidase (catalog number AP132P; Millipore, Burlington, MA, USA). The resulting signals were captured and measured using FluorChem SP imaging equipment (Alpha Innotech, San Leandro, CA, USA).

The sizes of the purified exosomes were assessed using a Zetasizer Nano-ZS DLS system (Malvern, Montréal, QC, Canada) in sets of three measurements. Each exosome sample was loaded into an ultraviolet microcuvette (BRAND; Essex, CT, USA) and examined at 25 °C. The analysis involved observing fluctuations in the intensity of the scattered light at a fixed angle of 173° and a wavelength of 633 nm. By measuring the Brownian motion of each particle, the peak of the Gaussian model fitted to the particle distribution was used to determine the average diameter of the exosomes, which is expressed as the PDI. Each data point represents the average of three automated measurements, with each measurement comprising 12–18 individual runs.

For TEM analysis, 10 μL of exosomes were fixed in 2.5% glutaraldehyde for 2 h. Fixed exosomes were then added to a 200 mesh Formvar coated with carbon and examined under a transmission electron microscope operated at 100 kV (HT-7700; Hitachi, Tokyo, Japan). Prior to imaging, the grids were stained with 2% uranyl acetate for 1 h.

### 2.5. Uptake of Fluorescently Labeled Exosomes by Macrophages

To track the internalization of exosomes by macrophages, Exo-Glow Exosome Labeling Kits (System Biosciences) were used to fluorescently label the purified exosomes before their application. These kits included Exo-Red and Exo-Green stains. The Exo-Red reagent uses acridine orange, a membrane-permeable dye that specifically labels single-stranded RNA present in exosomes. In contrast, the Exo-Green stain comprises a carboxyfluorescein succinimidyl diacetate ester, which emits green fluorescence when bound to the amino termini of intracellular proteins.

According to the manufacturer’s instructions, approximately 500 μL of the exosome suspension was mixed with 50 μL of either Exo-Red or Exo-Green and incubated for 10 min at 37 °C to allow for labeling. In this study, macrophages were subjected to overnight starvation before exosome treatment to enhance the cellular uptake of exosomes. The labeled exosomes were then incubated with primary cultured macrophages for 2 h and confocal microscopy images were captured using a FLUOVIEW FV10i microscope (Olympus, Tokyo, Japan).

### 2.6. Next-Generation Sequencing

RNA samples obtained from macrophages treated with 100 μg of CLP-exo for 24 h were used for NGS experiments. The results were compared with those of cells treated with Control-exo, and the experiments were conducted in duplicate (*n* = 2). The NEBNext Ultra Directional RNA Library Prep Kit (Illumina, San Diego, CA, USA) was used to construct NGS libraries following the manufacturer’s instructions. The Ribo-Zero ribosomal RNA (rRNA) Removal Kit (Illumina) was used to remove rRNA from the total RNA. The resulting rRNA-depleted samples were then subjected to fragmentation and reverse transcription. First-strand cDNA was synthesized using ProtoScript II Reverse Transcriptase, random primers, and actinomycin-D. Next, the second-strand cDNA was generated using the Second Strand Synthesis Enzyme Mix (Illumina) containing dACG-TP/dUTP.

Purified double-stranded cDNA was subjected to AxyPrep Mag PCR Clean-up (Axygen, New York, NY, USA), followed by treatment with End Prep Enzyme Mix. This treatment repaired both ends of the cDNA and added a dA-tail to one reaction, which was then ligated with adapters at both ends using TA ligation. The adaptor-ligated DNA fragments were selected to recover fragments of approximately 360 bp with an insert size of approximately 300 bp. The second strand, marked with dUTP, was digested using Uracil-Specific Excision Reagent from New England Biolabs (New England Biolabs, Ipswich, MA, USA).

Each sample underwent 11 cycles of PCR amplification using the P5 and P7 primers. These primers had sequences that enabled them to anneal to the flow cell during bridge PCR. In addition to the six-base index, the P7 primer enabled multiplexing. The resulting PCR products were purified using AxyPrep Mag PCR Clean-up (Axygen), analyzed using an Agilent 2100 Bioanalyzer (Agilent Technologies, Palo Alto, CA, USA), and quantified using a Qubit 2.0 Fluorometer (Invitrogen, Carlsbad, CA, USA). Following the manufacturer’s instructions, libraries with distinct indices were combined, multiplexed, and loaded onto an Illumina HiSeq instrument. The sequencing procedure was a 2 × 150 paired-end configuration. Image analysis and base calling were performed using the HiSeq Control Software (v2.2.68) and OLB with GAPipeline-1.6 (Illumina). Finally, GENEWIZ (Genewiz, South Plainfield, NJ, USA) processed and analyzed the sequences.

The data analysis process involved the utilization of Trimmomatic v0.30 (Illumina) to filter and obtain high-quality clean data in the FASTQ format. This included the removal of technical sequences such as adapters, PCR primers, and their fragments, and the elimination of bases with quality scores below 20. Reference genome sequences and gene model annotation files specific to the relevant species were acquired from the University of California, Santa Cruz genome website. Subsequently, the reference genome sequence was indexed using Hisat2 (v2.0.1) to facilitate alignment with clean data against the reference genome. Known gene feature format (gff) annotation files were converted to FASTA format, and the resulting transcripts were appropriately indexed. HTSeq v0.6.1 was used to estimate the expression levels of genes and isoforms based on clean paired-end data. Differential expression analysis was conducted using the DESeq Bioconductor package with Benjamini and Hochberg’s approach to control for false discovery rate. Genes with a *p*-value < 0.05 were considered to be differentially expressed. After the assembly of a transcriptome from one or more samples, the Cuffcompare tool within Cufflinks v2.2.1 was used to compare the assembly with known transcripts. RNA samples were quantified using a NanoDrop 2000 spectrophotometer (Thermo Fisher Scientific, Waltham, MA, USA) and a Qubit 2.0 Fluorometer (Thermo Fisher Scientific). The RNA quality was assessed by examining the Qubit/NanoDrop ratio and Caliper RNA Quality Score using The Caliper LabChip GX Electrophoresis System (Caliper Life Sciences, Hopkinton, MA, USA).

cDNA libraries were prepared using TruSeq Stranded Total RNA Sample Preparation Guide (catalog number 15031048; Illumina). Each library sample was pooled and sequenced using a NextSeq 500 (Illumina). For RNA-seq, row sequences were assessed for quality using FastQC v0.11.8. and trimmed for primer-adaptor sequences using the RNA-seq alignment tool from BaseSpace (Illumina), followed by alignment with the human reference genome (hg38) using STAR v2.7.3a. Expression levels and differential expression analyses were performed using DESeq2 v1.24.0. (selection criteria: *p*-adj value < 0.05, |fold-change| > 2).

### 2.7. Real-Time Polymerase Chain Reaction

For the real-time PCR experiments, macrophages were exposed to either 100 μg of CLP-exo or Control-exo for 24 h (*n* = 6 for each group). Total RNA was extracted from the collected cells using RNeasy Mini Kits (catalog number 217004; Qiagen, Hilden, Germany), and the RNA concentration was determined using an SSP-3000 NanoDrop spectrophotometer (Infinigen Biotech, City of Industry, CA, USA). RNA was converted into cDNA using a High-Capacity cDNA Reverse Transcription Kit (catalog number 4368814; Applied Biosystems, Foster City, CA, USA). TaqMan Universal PCR Master Mix (No UNG, PN 4324018, Applied Biosystems) and specific primers were used to amplify the cDNA. Alternatively, Power SYBR Green PCR Master Mix (catalog number 4367659; Applied Biosystems) with specific primers was used for cDNA amplification.

These primers included the selected abundantly expressed lncRNAs, identified from the NGS experiments for macrophages, and MIR17HG-related genes, identified from the combined approach of the intersection of the upregulated mRNAs in the NGS experiments and the explored MIR17HG-related genes from the MEM website (https://biit.cs.ut.ee/mem/, accessed on 14 November 2022), providing gene expression similarity searches for large collections of microarray datasets [4].

The detected lncRNAs included MIR17HG (forward: 5′-AGAAACATAGTCAC-TTCTGACTCCATTCTT-3′; reverse: 5′-AACGTAGCATTCAATCCGTAACACT-3′); Crnde1 (forward: 5′-TCTGGTTGTCACCTCCGCTCTAC-3′; reverse: 5′-TGCTAATGATCAGCTTTCGCCTTCC-3′); Crnde2 (forward: 5′-TGG-GAGCCTCTGTAGCGT-3′; reverse: 5′-TCCATAGCAATGTCTGCCACTGTGA-3′); Junos (forward: 5′-GGGAGCTGACTTCTGAGTATATC-3′; reverse: 5′-GTT-GACAATCAATAACTCTTTGAGGTAG-3′); AI480526 (forward: 5′-CCATTCTCTCTGAACCTACAAAC-3′; reverse: 5′-CTCAGTCAAATTGGGCCT-3′); C130046K22Rik (forward: 5′-TTTATGTCCCAAGAGACTGCC-3′; reverse: 5′-TAAGGACTTTCTCAATAGACAGCGA-3′); Gm8066 (forward: 5′-GCG-GAGCTGTTTCACCA-3′; reverse: 5′-AAAGAAGCAGCTATGCCTAAG-3′), Gm10863 (forward: 5′-TGCATGTAATACCAAAGGCAAC-3′; reverse: 5′-GAGCCAGC-TATGGTGACT-3′); Gm14221 (forward: 5′-TTGTCCCTCCTCCTATCTCTAAG-3′; reverse: 5′-CTAGGCAATAGATGATGGAGAACTAAC-3′); Gm15608 (forward: 5′-CCCATGTCACATGAAATTGAGTAAG-3′; reverse: 5′-CCATCTGAAACTAC-CTAGTGTTCT-3′); Gm16278 (forward: 5′-GACAGCCAGGGCTATACAGA-3′; reverse: 5′-AGATGGTCAGTCACCGGA-3′); Gm20186 (forward: 5′-CTCCTTAG-TTACGTAGGTTCCTTGATA-3′; reverse: 5′-TAGAGGAGTCTCGACGGT-3′); and Gm49654 (forward: 5′-AGACCCAATCCTGGCTC-3′; reverse: 5′-ATTGG-TAGGCCCTTGCTT-3′).

Five representative MIR17HG-related mRNAs included Cdca7 (forward: 5′-TGTGGTCCCTGCCTTCGAAAC-3′; reverse: 5′-TGATACTTTGCCAGATACACCAGGACT-3′), Qser1 (forward: 5′-CAATTTAGTTTGTTGCCTTCAACACTTGG-3′; reverse: 5′-TTGCACTTGATAGGTAGCTATGTAAGGGAT-3′); Nup153 (forward: 5′-CCAGCAATAGGAACTTGGGATTGTGATAC-3′; reverse: 5′-GAAGAGGAAGCAGTCACAGGACTTT-3′); Dbf4 (forward: 5′-TCATGCTTCTGACCTGGTGGC-3′; reverse: 5′-CTAATAGCATTCTCACTTTCCGCTGGAT-3′); and Flvcr1 (forward: 5′-TCAGCCTTTACTCGCTGGTGAAC-3′; reverse: 5′-TGGCCGGGAAGATGAGGG-3′). The primers for mGAPDH were forward: 5′-GGAGAGTGTTTCCTCGTCCC-3′ and reverse: 5′-ATGAAGGGGTCGTTGATGGC-3′.

Real-time PCR was performed using the 7500 Fast Real-Time PCR system (Applied Biosystems) with Power SYBR Green PCR Master Mix (catalog number 4367659; Applied Biosystems). The expression level of the genes at the transcription level was quantified using the 2^−ΔΔCt^ method with normalized cycle threshold (Ct) values, where m-GAPDH served as the internal control. Statistical significance in the expression of transcripts was determined when the mean value of all samples differed by at least two-fold compared with the control, with a *p*-value < 0.05.

### 2.8. Knockdown of MIR17HG in Macrophages

To suppress MIR17HG gene activity in macrophages, the cells were transfected with 25–50 nM siRNA specifically targeting mouse MIR17HG (si-MIR17HG) or 25 nM control siRNA (scrambled siRNA) for 6 h (*n* = 6 for each group). Following a change in the culture medium, the macrophages were subjected to CLP-exo treatment for 24 h. Transfection was performed using Lipofectamine RNAiMAX (catalog number 13778; Invitrogen). The siRNA sequence for targeting MIR17HG pool was siRNA-1: 5′-GGAUUUAGUAAGAAGUUGU-3′; siRNA-2: 5′-UCAUUGGAGUGAAACCUAA-3′; siRNA-3: 5′-AAUAGAACAUUGUGAGGUA -3′; siRNA-4: 5′-AGGUUUAAGCUGACACUAU-3′. The siRNA sequence for Scramble siRNA pool was siRNA-1: 5′-UGGUUUACAUGUCGACUAA-3′; siRNA-2: 5′-UGGUUUACAUGUUGUGUGA-3′; siRNA-3: 5′-UGGUUUACAUGUUUUCUGA-3′; siRNA-4: 5′-UGGUUUACAUGUUUUCCUA-3′. The MIR17HG expression level was determined using real-time PCR following previously described methods.

### 2.9. Cell Proliferation

Cell proliferation was assessed using the CytoScan WST−1 Cell Proliferation Assay (catalog number ab65475; Abcam), a method in which metabolically active cells reduce the WST−1 reagent, resulting in the production of a colored formazan product. Macrophages were plated in a 96-well plate, treated with 100 μg of CLP-exo, and exposed to si-MIR17HG or scrambled siRNA (*n* = 6 for each group). The cells were allowed to grow, and after a 4 h incubation period, the cell culture medium was replaced with WST−1 reagent and incubated with the cells. After 24 h, the microplate reader (Thermo Fisher Scientific) with a spectrophotometric function, set at a wavelength of 450–490 nm, was used to measure the absorbance of the formazan product. The number of viable cells was determined based on the intensity of color, which directly corresponded to cell viability. 

### 2.10. Activated Signal Pathway of Macrophages Treated with CLP-Exo

To explore the signaling pathways implicated in macrophages during CLP-exo treatment, the Cignal Finder Reporter Array (catalog number CCA-901L; Qiagen) was employed. This array facilitated the identification of 45 distinct signal transduction pathways. In a 96-well plate, duplicate copies of the 45 inducible transcription factor-responsive constructs regulated the expression of the firefly luciferase reporter gene. To normalize the transfection efficiency, the Renilla luciferase gene was introduced into the wells. The constructs were reverse-transfected into macrophages at a density of 1 × 10^5^ cells per well using the Effectene Transfection Reagent (catalog number 301425; Qiagen), along with Opti-MEM medium (catalog number 31985062; Invitrogen), supplemented with 10% fetal bovine serum and 1% non-essential amino acids (catalog number 11140-050; Invitrogen).

Following the manufacturer’s instructions, the Dual-Glo Luciferase Assay System (catalog number E2940; Promega, Madison, WI, USA) was used to measure luciferase activity in macrophages 24 h after treatment with CLP-exo or Control-exo. Luminescence was quantified using a Hidex Sense microplate reader (Turku, Finland). A comparison was made between the luminescence measurements of cells treated with CLP-exo and those treated with Control-exo (*n* = 6 for each group). The arrays included both negative and positive luminescence controls. A signaling pathway was considered significantly activated when there was a 1.5-fold increase in relative luminescence, with a *p*-value < 0.01.

### 2.11. Detection of NF-κB Activation

The NF-κB pathway activation was evaluated in primary cultured macrophages isolated from B10.Cg-H2k Tg(NFkB/Fos-luc)26Rinc/J mice. Macrophages were treated with 100 μg of CLP-exo or Control-exo. For positive and negative controls, 25 μg/mL LPS [45,46] or PBS was administered, respectively (*n* = 6 for each group). Luminescence was measured at 2, 4, and 6 h post treatment using a Hidex Sense microplate reader. A signaling pathway was considered significantly activated when there was a 1.5-fold increase in luminescence, with a *p*-value < 0.01.

### 2.12. Extraction of Exosomal Protein

The exosomal proteins found in CLP-exo and Control-exo were extracted using the T-PER tissue protein extraction reagent (catalog number 78510; Thermo Fisher Scientific). To eliminate impurities, protein samples underwent desalting using Amicon Ultra-15 (Millipore) and were subsequently quantified using the BCA protein assay (catalog number 23225; Thermo Fisher Scientific). The obtained protein samples were subjected to proteomic analysis using isobaric tag labeling for relative and absolute quantitation (iTRAQ).

### 2.13. iTRAQ Proteomic Analysis of the Exosomal Content

For iTRAQ labeling, the protein samples (25 µg) were dried using SpeedVac and reconstituted in iTRAQ dissolution buffer, containing 0.5 M pH 8.5 triethylammonium bicarbonate. The samples in duplicate were reduced with iTRAQ reduction buffer tris-2-carboxyethyl phosphine at 60 °C for 30 min, followed by alkylation in the dark using iodoacetamide at 37 °C for the same duration. After protein digestion with sequencing-grade modified trypsin (V511A; Promega), the resulting peptides were dried and reconstituted in iTRAQ dissolution buffer. They were then labeled using iTRAQ labeling reagents, according to the manufacturer’s instructions (Applied Biosystems).

iTRAQ-labeled samples were analyzed using a Q Exactive HF mass spectrometer (Thermo Fisher Scientific) coupled to an UltiMate 3000 RSLCnano HPLC System (Thermo Fisher Scientific). The labeled peptides were pooled, desalted using Sep-Pak C18 cartridges (Waters, Milford, MA, USA), and dried. The desalted peptides were resuspended in 0.5% trifluoroacetic acid and loaded onto an EASY-Spray C18 column (Thermo Fisher Scientific). Separation was achieved using a solution of 0.1% formic acid in varying acetonitrile proportions (5–80%). The top 15 most abundant precursor ions within the 375–1400 *m*/*z* scan range were selected for fragmentation in high collision dissociation mode, with the normalized collision energy set to 33 ± 1%. In the full MS scan, the resolution was set to 60,000 at 200 *m*/*z*, AGC target to 3 × 10^6^, and maximum injection time to 50 ms. For the MS/MS scan, the resolution was set to 15,000, AGC target to 5e4, and maximum injection time was set to 100 ms. Dynamic exclusion of the selected precursor ions was implemented with a 20 s time window.

The Mascot search algorithm (version 2.5, Matrix Science, London, UK) was used to analyze raw MS data. The search was conducted against the Swiss-Prot human protein database using Proteome Discoverer software (version 2.1; Thermo Fisher Scientific). The search parameters specified carbamidomethylation at cysteine as the fixed modification and oxidation at methionine, acetylation at the protein N-terminus, iTRAQ labeling at the peptide N-terminus, and lysine residues as the dynamic modifications. The tolerance for MS and MS/MS was set to 10 ppm and 0.02 Da, respectively, and a maximum of two missed cleavage sites were allowed.

### 2.14. RNA Pull-Down Assay

A pull-down assay was performed to detect physical interactions between proteins, confirming predicted protein–protein interactions and probing protein-binding partners [47]. In this study, RNA pull-down assays were performed to investigate protein binding to MIR17HG. For in vitro linear synthesis of MIR17HG, a circular plasmid, pBluescript KSII, was used along with the TranscriptAid T7 High Yield Transcription Kit (Thermo Fisher Scientific, Waltham, MA, USA). Following in vitro transcription, MIR17HG RNA and negative control RNA (provided in the kit) were labeled with biotin using the Pierce RNA 3′ end desthiobiotinylation kit (Thermo Fisher Scientific).

The Thermo Pierce Magnetic RNA-Protein Pull-Down Kit (Thermo Fisher Scientific) was used to pull-down the RNA-protein complexes according to the manufacturer’s instructions. In this process, 50 pmol of biotinylated negative control RNA and MIR17HG were captured using 50 μL of streptavidin magnetic beads. These complexes were then allowed to interact with 200 μg of lysate extracted from the macrophages. Elution was performed to release the RNA-binding protein complexes, which were further analyzed using LC-MS/MS and Western blotting.

After the RNA pull-down assays, the proteins obtained were digested using sequencing-grade modified trypsin (V511A; Promega) for LC-MS/MS analysis. Following trypsin digestion, the resulting peptide samples were analyzed using a Q Exactive HF mass spectrometer (Thermo Fisher Scientific) in conjunction with an UltiMate 3000 RSLCnano HPLC System (Thermo Fisher Scientific). Tryptic peptides were desalted using Sep-Pak C18 cartridges (Waters).

To validate the expression of selected protein targets from the pull-down proteins, Western blotting was performed on the input protein samples from macrophage cell lysates, the pulled-down proteins by MIR17HG, and the pulled-down proteins by control RNA primary antibodies against MBNL1 (catalog number ab45899, 1:1000; Abcam), MBNL2 (catalog number ab222242, 1:1000; Abcam), and RAC2 (catalog number ab154711, 1:1000; Abcam) were used in the Western blotting analysis, following the aforementioned procedures.

### 2.15. Construction of Interaction Network

Separate interaction networks were constructed to depict the exosomal protein network within CLP-exo and the proteins were obtained through pull-down experiments. These networks illustrate the interconnections among the identified targets. GeneMANIA (http://www.genemania.org/), an established online tool for discerning internal correlations within gene sets [48], was used to establish the interaction networks. The GeneMANIA network incorporates various types of interactions including physical interactions, co-expression patterns, predicted associations, co-localization, genetic interactions, pathway relationships, and shared protein domains.

### 2.16. Statistical Analysis

All statistical analyses in this investigation were conducted using SPSS version 23.0 for Windows (IBM Inc., Chicago, IL, USA). The Kolmogorov–Smirnov test was used to evaluate the normalization of the dispersed data for continuous variables. The unpaired Student’s *t*-test was employed to evaluate continuous data that followed a normal distribution, whereas the Mann–Whitney U test was utilized for continuous data that did not follow a normal distribution. The results are presented as their mean with the error bar indicating the standard error of the mean. Significance was defined as *p* values that were less than 0.05.

## 3. Results

### 3.1. Characterization of the Purified Exosomes

**Figure 1:**
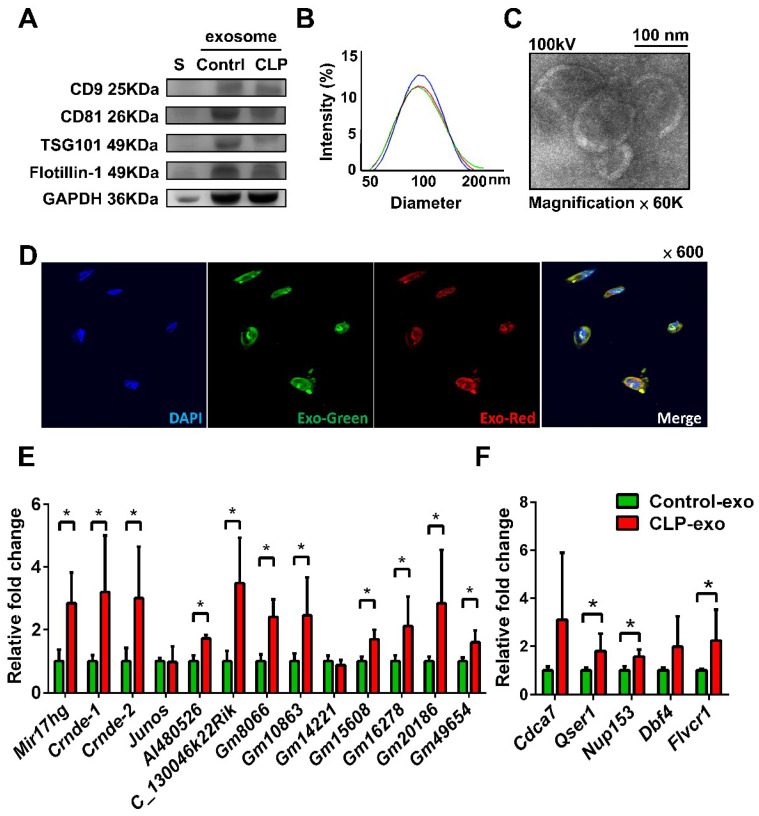
Characterization of purified exosomes using (**A**) Western blotting for exosomal surface markers of the purified circulating exosomes in septic mice (CLP-exo) and sham-operated mice (Control-exo), with medium (S) as the control, (**B**) dynamic light scattering (DLS) for measuring particle diameter and size distribution in triplicate with different color lines indicating different measurements, and (**C**) transmission electron microscopy (TEM) analyses. (**D**) After incubating the exosomes with the macrophages for 2 h without a transfection reagent, the uptake of fluorescently labeled single-stranded RNAs and proteins of CLP-exo into primary cultured macrophages was observed through Exo-Red and Exo-Green staining, respectively. (**E**) Real-time PCR of the 12 most abundantly expressed lncRNAs from the next-generation sequencing (NGS) experiments comparing macrophages treated with CLP-exo versus Control-exo. (**F**) Real-time PCR of five MIR17HG-related transcripts selected by the combined approach of the intersection of those upregulated mRNAs in the NGS experiments and the explored MIR17HG-related genes from the MEM website. *, a *p*-value < 0.05 with at least 2-fold expression.

Western blot analysis revealed that the purified exosomes obtained from the cecum ligation and puncture (CLP)-exo and Control-exo samples had significantly higher levels of positive exosomal surface markers, including CD9, CD81, TSG101, and Flotillin-1 than those from the control medium (Figure 1A). The size distribution of the exosomes was assessed using dynamic light scattering (DLS), indicating an average size of 102.2 ± 38.8 nm with a single peak distribution and a polydispersity index (PDI) of approximately 0.79 (Figure 1B). Transmission electron microscopy (TEM) images captured at ×60,000 magnification demonstrated that the exosomes possessed a circular shape and lipid bilayers and exhibited a size range and morphology within acceptable parameters (Figure 1C). Overall, the quality of the purified exosomes was excellent.

The uptake of fluorescently labeled single-stranded RNAs and proteins from both Control-exo and CLP-exo into primary cultured macrophages was observed through Exo-Red and Exo-Green staining, respectively, after the incubation of the exosomes with macrophages for 2 h without a transfection reagent (Figure 1D). The dysregulated transcripts obtained from duplicate next-generation sequencing (NGS) experiments comparing macrophages treated with CLP-exo and Control-exo are listed in Supplementary File S1. Among these, 12 lncRNAs (*MIR17HG*, *Crnde*, *Junos*, *AI480526*, *C130046K22Rik*, *Gm8066*, *Gm10863*, *Gm14221*, *Gm15608*, *Gm16278*, *Gm20186*, and *Gm49654*) were abundantly expressed. To validate these findings, real-time PCR was performed, which confirmed a consistent overexpression pattern for most of the selected lncRNAs (Figure 1E). Furthermore, real-time PCR revealed the significant overexpression of three MIR17HG-related mRNAs (*Qser1*, *Nup153*, and *Flvcr1*) of five representative MIR17HG-related genes identified through a combined approach involving the intersection of upregulated mRNAs from NGS experiments and MIR17HG-related genes from the Multi Experiment Matrix (MEM) website [9] (Figure 1F). The expression of *Cdca7* and *Dbf4* transcripts in macrophages treated with CLP-exo was higher than that in macrophages treated with Control-exo, although this difference was not statistically significant.

### 3.2. The Effect of MIR17HG Knockdown on Cell Proliferation and Lipid Peroxidation

**Figure 2:**
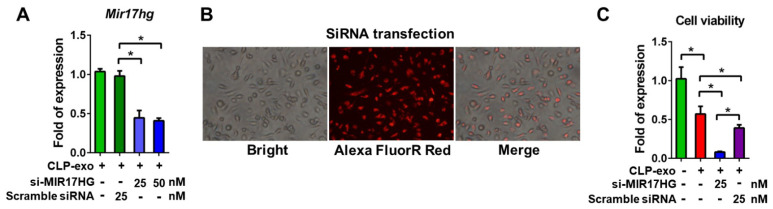
(**A**) Following transfection of si-MIR17HG into macrophages for 6 h, reduction in MIR17HG mRNA expression was found in these macrophages treated with 100 µg CLP-exo for 24 h, while the reduction was not found in those CLP-exo-treated macrophages transfected with 25 mM scrambled siRNA. (**B**) Successful transfection of si-MIR17HG combined with Alexa Fluor Red could be visualized in macrophages under confocal microscopy. (**C**) The proliferation detected by WST−1 cell proliferation assay after 24 h in the cultured macrophages receiving CLP-exo treatment in the presence or absence of 25 nM si-MIR17HG or scrambled siRNA. *, a *p*-value < 0.05 with at least 2-fold expression.

Transfection of small interfering RNA (siRNA) targeting MIR17HG (si-MIR17HG) into macrophages treated with CLP-exo was successfully achieved using an effectene transfection reagent. At concentrations of 25 and 50 nM, si-MIR17HG significantly reduced MIR17HG mRNA expression compared with that in macrophages transfected with 25 mM scrambled siRNA (Figure 2A). The successful transfection of si-MIR17HG combined with Alexa Fluor Red was visualized in macrophages using confocal microscopy (Figure 2B). The WST−1 cell proliferation assay revealed that CLP-exo treatment significantly decreased the proliferation of primary cultured macrophages (Figure 2C); however, the inhibition of MIR17HG by si-MIR17HG significantly reduced cell viability in CLP-exo-treated macrophages compared with those transfected with scrambled siRNA (Figure 2C). 

### 3.3. Involvement of NF-κB Pathway in the Induction of MIR17HG

**Figure 3:**
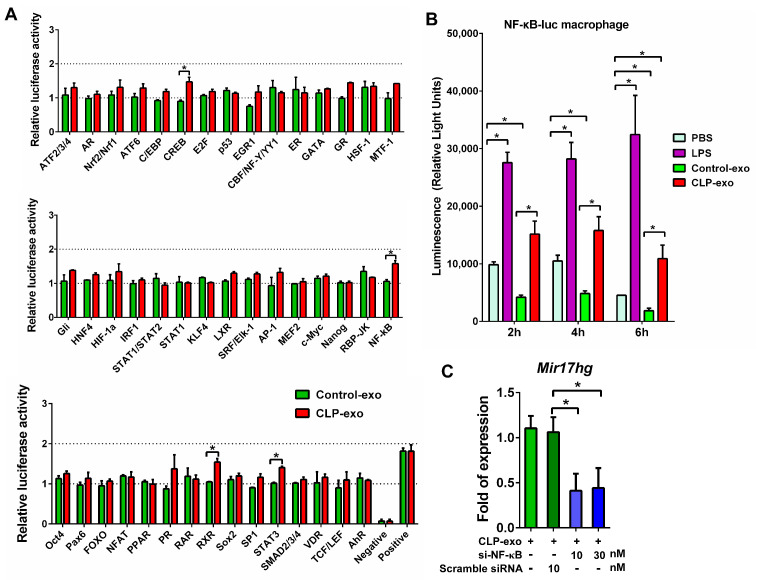
(**A**) Cignal Finder Reporter Arrays were used to assess activated signal transduction pathways in macrophages 24 h after the uptake of CLP-exo, in comparison with those treated with Control-exo. The Reporter Array comprised 45 constructs that respond to inducible transcription factors and regulate the expression of the firefly luciferase reporter gene. The assay was performed in duplicate in a 96-well plate, and the Renilla luciferase gene was included for transfection efficiency normalization. (**B**) Luminescence expression was measured in primary cultured macrophages containing a luciferase gene driven by two copies of the NF-κB regulatory element. The macrophages were treated with 100 μg of CLP-exo, 100 μg of Control-exo, 25 μg/mL of LPS as a positive control, or PBS as a control. Luminescence measurements were taken at 2, 4, and 6 h using a Hidex Sense microplate reader. (**C**) To assess the impact on MIR17HG transcripts expression, si-NF-κB at concentrations of 10–30 nM was transfected into macrophages for 6 h. The transfection significantly reduced MIR17HG transcript expression in macrophages treated with 100 μg of CLP-exo for 24 h, compared with macrophages transfected with scrambled siRNA. The statistical significance of luminescence and MIR17HG transcript expression is denoted by “*”, indicating a *p*-value < 0.01 with at least a 1.5-fold expression for luminescence and a *p*-value < 0.05 with at least a 2-fold expression for MIR17HG transcript expression.

Compared with macrophages treated with Control-exo, the uptake of CLP-exo by macrophages for 24 h resulted in the activation of several signal transduction pathways, including cAMP response element-binding protein (CREB), retinoid X receptor (RXR), signal transducer and activator of transcription 3 (STAT3), and nuclear factor-kappa B (NF-κB), as observed in the Cignal Finder Reporter Arrays (Figure 3A). These arrays monitor 45 pathways regulated by inducible transcription factors.

NF-κB pathway activation was specifically confirmed in primary cultured macrophages carrying a luciferase gene driven by two copies of the regulatory element of NF-kB. Macrophages treated with 100 μg of CLP-exo exhibited significantly higher luminescence than those treated with Control-exo at 2, 4, and 6 h (Figure 3B). Furthermore, compared with macrophages treated with phosphate-buffered saline (PBS), CLP-exo treatment induced significant NF-κB activation at 6 h, while Control-exo treatment resulted in a reduction in NF-κB activation within 6 h.

Additionally, the transfection of si-NF-κB into macrophages for 6 h significantly decreased the expression of MIR17HG transcripts in macrophages treated with CLP-exo for 24 h, in comparison with macrophages transfected with scrambled siRNA beforehand (Figure 3C).

### 3.4. iTRAQ Proteomic Analysis and Interaction Network of Exosomal Proteins in CLP-Exo

**Figure 4:**
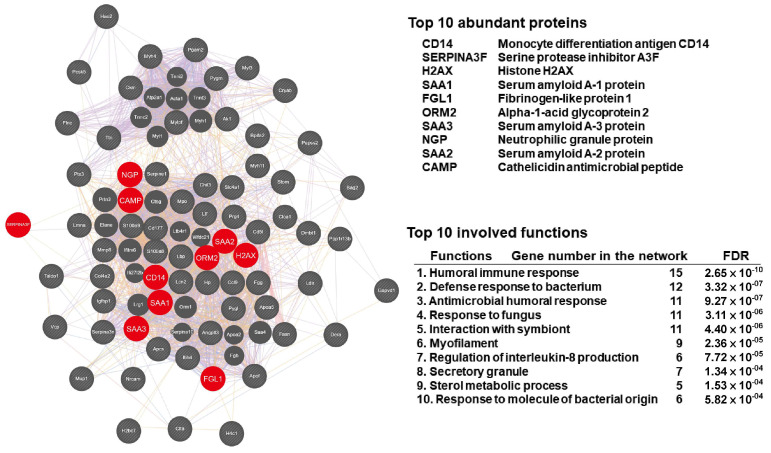
The interaction network of these 70 dysregulated proteins was reconstructed using GeneMANIA. The visualization included the ten most abundant proteins (highlighted in red) found within CLP-exo and the top ten associated functions of these dysregulated proteins.

Duplicate iTRAQ proteomic analysis was conducted to examine the exosomal protein content of CLP-exo and Control-exo. The analysis revealed 70 proteins that were dysregulated by more than 2-fold, with 67 upregulated and 3 downregulated proteins (Supplementary File S2). The most abundant proteins detected in CLP-exo were CD14, SERPINA3F, H2AX, SAA1, FGL1, ORM2, SAA3, NGP, SAA2, and CAMP (Figure 4). GeneMANIA was used to reconstruct the interaction network of these 70 dysregulated proteins, and the top associated functions are presented in Figure 4. These functions include humoral immune response, defense response to bacteria, and antimicrobial humoral response ranking among the top three functions. Additionally, the upregulation of the exosome surface markers CD9 and CD81 was observed among the upregulated proteins.

### 3.5. Proteomic Analysis of MIR17HG Pull-Down Proteins

**Figure 5:**
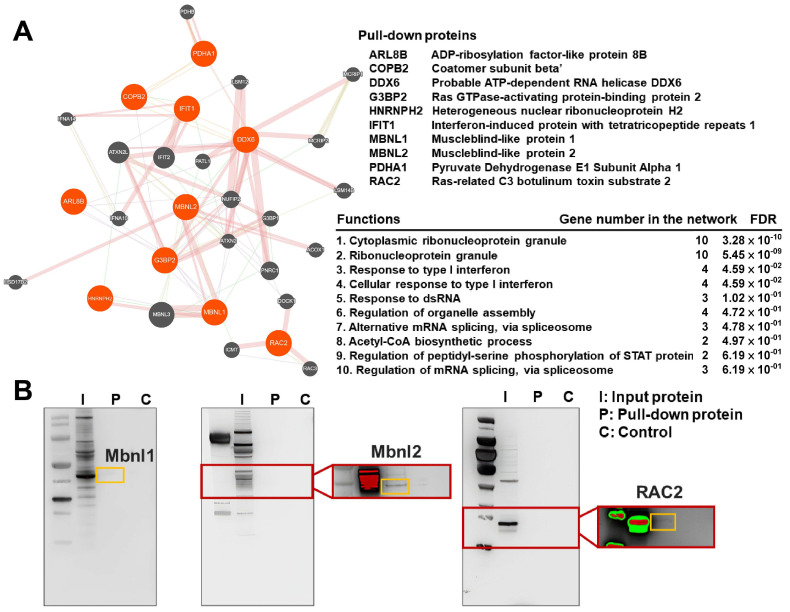
(**A**) GeneMANIA was used to reconstruct the interaction network of ten proteins pulled down by MIR17HG. The top 10 functions associated with these proteins were presented. (**B**) To validate their presence in the MIR17HG pull-down proteins, Western blotting was performed on three selected representative targets (Mbnl1, Mbnl2, and RAC2), which were highlighted in the yellow boxes.

RNA pull-down assays were used to identify the proteins that bind to MIR17HG. A subsequent LC-MS/MS analysis of these pull-down proteins resulted in the identification of 10 protein targets: ARl8B, COPB2, DDX6, G3BP2, HNRNPH2, IFIT1, MBNL1, MBNL2, PDHA1, and RAC2 (Figure 5A). Western blotting was performed on three selected representative targets (MBNL1, MBNL2, and RAC2) to confirm their presence among the MIR17HG pull-down proteins (Figure 5B). The interaction network of these ten pull-down proteins was reconstructed using GeneMANIA, revealing the top ten associated functions to be cytoplasmic ribonucleoprotein granules, ribonucleoprotein granules, response to type I interferon, cellular response to type I interferon, response to double-stranded RNA, the regulation of organelle assembly, alternative mRNA splicing via the spliceosome, acetyl coenzyme A (acetyl-CoA) biosynthetic process, the regulation of peptidyl-serine phosphorylation of STAT protein, and the regulation of mRNA splicing via the spliceosome.

## 4. Discussion

This study demonstrated that the circulating exosomes during sepsis induced the activation of NF-κB/MIR17HG in the macrophages and the inhibition of MIR17HG expression led to significantly decreased cell viability. MIR17HG is involved in cell survival, proliferation, and differentiation [49]. The cell proliferation is induced by MIR17HG via the upregulation of miR-17-92 [50], an miRNA cluster encoded by MIR17HG [50,51,52]. The miR-17-92 cluster is essential for immune system development and plays a role in controlling the cells engaged in innate and adaptive immunity [51]. Moreover, the miR-17-92 cluster plays a role in regulating biological processes such as oncogenesis [53,54,55,56], neurogenesis [57,58], immune system modulation [51,59], and the pathogenesis of osteoarthritis [50,60] and immunoglobulin A nephropathy [61]. A growing body of evidence suggests that MIR17HG is involved in promoting CD34+ hematopoietic progenitor cell differentiation into monocytes via a macrophage colony-stimulating factor [62].

In sepsis, NF-κB is activated by different stimuli, such as toll-like receptor ligands and cytokines, with a downstream induction of pro-inflammatory genes. In this study, the transfection of si-NF-κB into macrophages significantly decreased the upregulation of MIR17HG in macrophages upon CLP-exo treatment. However, NF-κB and miR-17-92 have inter-related regulatory functions. NF-κB signaling can influence miR-17-92 expression, and in turn, the miR-17-92 cluster can modulate the activity or expression of components within the NF-κB pathway. For example, tumor necrosis factor alpha (TNFα) induces miR-17-92 expression in an NF-κB-dependent manner [63], whereas miR-17-92 negatively regulates the NF-κB signaling pathway by directly targeting tumor necrosis factor receptor-associated factor 3 or TNF alpha-induced protein 3 [64]. There is evidence that miR-223, miR-15a, and miR-16 play roles in controlling NF-κB signaling as macrophages differentiate [65]. Notably, in this study, the uptake of CLP-exo by macrophages for 24 h resulted in the activation of NF-κB and STAT3. STAT3 [66] and multiple other transcription factors, including STAT5 [67], c-Myc [68,69], MYCN [70], MYB [71], Spi-1 and Fli-1 [72], Pim-1 [68], cyclin D1 [73], AML1 [74], and E2F family members [68], activate the transcription of MIR17HG. 

In this study, the presence of three selected representative targets (MBNL1, MBNL2, and RAC2) was confirmed among the MIR17HG pull-down proteins. MBNL is a crucial RNA processing factor for the splicing activity in mRNA metabolism [75,76,77]. When coexpressed, MBNL1 and MBNL2 bind to the same RNA motifs with different affinities [78]; in particular, MBNL1 has the highest affinity for abundant cytoplasmic granules in response to stress [75]. The upregulation of RAC2 in macrophages during inflammation determines the signaling specificity that induces M2 differentiation [79] and podosome development [80]. In peritoneal inflammation, RAC2 is also indispensable for the accumulation of macrophages with effective phagocytosis [81]. The results revealed that the ten pull-down proteins of MIR17HG were mainly involved in ribonucleoprotein granules, type I interferons, the regulation of organelle assembly, the biosynthesis of acetyl-CoA, STAT protein phosphorylation, and mRNA splicing. Ribonucleoprotein granules control the localization, translation, stability, and degradation of specific mRNAs in response to cellular signals [82,83]. When cells encounter viral infections or other conditions that generate double-stranded RNA, they activate a defense mechanism known as the interferon response. Type I interferons are known for their potent antiviral activity, immunomodulatory effects on inflammatory responses, action as signaling molecules that promote communication between different immune cells, and tissue repair and regeneration processes [84,85,86]. The regulation of organelle assembly refers to the processes and mechanisms that control the formation, organization, and dynamics of cellular organelles [87,88]. Acetyl-CoA biosynthesis involves the synthesis of acetyl-CoA from various precursor molecules within the cell. Acetyl-CoA is a central metabolite involved in several cellular processes, including energy production, lipid synthesis, and the production of certain neurotransmitters [89,90]. mRNA splicing removes introns, connects exons, and generates mature mRNA molecules ready for translation, and is a fundamental process in eukaryotic gene expression [91,92]. 

Notably, this study focused on the analysis of pull-down proteins of MIR17HG. However, lncRNAs function through diverse mechanisms such as chromatin remodeling, gene expression regulation, and protein interaction modulation [13,15]. They can act as scaffolds, guiding the assembly of protein complexes, or as guides targeting specific proteins to specific genomic regions [13,14,15]. LncRNAs can also act as molecular sponges, sequestering microRNAs or RNA-binding proteins, thereby regulating gene expression [13,14,15]. Therefore, the functions of MIR17HG may not be limited to the aforementioned functions. However, further studies are needed to validate this function.

In this study, abundant proteins were identified inside exosomes. Among these identified proteins, CD14 confers lipopolysaccharide (LPS)-responsiveness to cells and the subsequent toll-like receptor (TLR) signaling pathway [93,94]; SERPINA3F exerts an inhibitory function in cytokine production [95] and is upregulated in apoptosis [96,97]; H2AX is a variant of the histone protein H2A, which is a component of the nucleosome, the basic unit of chromatin. H2AX is essential for the response to DNA damage and the preservation of genome stability [98,99]. This circulating histone family also regulates the acute lung injury induced by sepsis [100,101]. The SAA protein family is typically found in various inflammatory diseases and sepsis [102,103,104,105], particularly in exosomes [103,105]. For instance, exosomal SAA1 has been shown to be effective in treating lung injury caused by sepsis [105,106]. Exosomal SAA3 is associated with the induction of STAT3 signaling [107] and anti-inflammatory M2 polarization [108]; FGL-1 binds to and activates LAG-3, a regulatory protein on T cells, for immunotherapy [109,110] and anti-inflammation [111]; ORM2, also known as AGP2, can inhibit neutrophil migration in diabetic mice with sepsis [112]; NGP, specific for mouse neutrophils [113], exhibits sequence similarity to an antimicrobial protein CAMP [113] and demonstrates inflammation modulation [113,114]. Notably, NGP interacts with the LPS–LBP complex [114,115], inhibiting its binding to toll-like receptor 4 (TLR4) and subsequent inflammatory signaling [114]. Overall, these exosomal proteins may play a role in mediating various biological functions and the pathogenesis of sepsis.

There are several restrictions on this study. First, the results could be affected by the severity of sepsis. The conventional CLP method may entail a mid-grade form of sepsis, in which the ligation covers 50% of the cecum, or a high-grade form of sepsis, in which 75% of the cecum is ligated. Mid-grade sepsis would result in a 40% survival rate, but all high-grade sepsis animals died within 4 days following CLP induction [44]. In all severity ratings, sepsis begins around 12 h after CLP induction, with the majority of mortality occurring within the first 48 h [110]. The findings of this study are considerably more similar to those of previous studies on high-grade sepsis, while they may not be generalizable to settings of varying severities of sepsis. Second, the time-dependent progression of MIR17HG expression and its dynamic impact were not investigated, potentially imposing a constraint on the interpretation of the findings. Third, during sepsis, many circulating immune cells are important in different phases of the immune response. In the early stages of sepsis, neutrophils are among the first to arrive at the site of infection, are often more prominently involved, and are crucial for the phagocytosis of invading pathogens and help contain the infection at the initial site. Macrophages, on the other hand, play a role in the later stages of the immune response. In addition, dendritic cells in sepsis play a crucial role by capturing and presenting pathogens to initiate immune responses, regulating the balance between pro-inflammatory and anti-inflammatory reactions, and acting as a bridge between the innate and adaptive immune systems, helping to coordinate the body’s defense against the infection and prevent excessive inflammation. They are involved in the cleanup and resolution phase of inflammation, helping to resolve the inflammatory response and promote tissue repair. The relative importance of these immune cells can vary depending on the specific circumstances and severity of the septic condition. However, this study focused on the study and did not provide the data regarding the role of neutrophils or dendritic cells. Fourth, the study of macrophage function other than cell viability may provide important information. Our yet to be published data had shown that CLP-exo treatment for 24 h significantly inhibited the phagocytic function of macrophages compared with those treated by Control-exo; however, the role of MIR17HG in macrophage phagocytosis is not explored in this study. Experimental investigation is necessary to demonstrate the function of MIR17HG pull-down proteins, including Mbnl1, Mbnl2, and RAC2. This would be the next step in the research.

In conclusion, this study found significant MIR17HG overexpression in macrophages treated with circulating exosomes from mice with sepsis. The results of this study could potentially have a significant impact on sepsis research, particularly in understanding the role of exosomes in regulating inflammatory responses and mediating pathogenic pathways in macrophages. It is acknowledged that while this study provides valuable insights into the role of exosomes and MIR17HG expression in macrophages during sepsis, further research is needed to fully understand the relationship between these findings and the varying severities of sepsis. This includes investigating the time-dependent changes in MIR17HG expression, its impact on different immune cells, and how these vary in mid-grade versus high-grade sepsis. Further investigation into the precise mechanism of action of exosomes in macrophage activation may provide a new avenue for research on sepsis treatment.

## 5. Conclusions

Circulating exosomes derived from polymicrobial sepsis contain various non-coding RNAs and proteins. The treatment of macrophages by the circulating exosomes obtained from the blood of septic mice induce NF-κB/MIR17HG pathway activation. The construction of interaction networks of the pull-down proteins of MIR17HG revealed that they were mainly involved in ribonucleoprotein granules, type I interferons, the regulation of organelle assembly, the biosynthesis of acetyl-CoA, STAT protein phosphorylation, and mRNA splicing. In addition, the inhibition of MIR17HG expression by RNA interference significantly decreased cell viability.

## Data Availability

Not applicable.

## Supplementary Materials

The following supporting information can be downloaded at: https://www.mdpi.com/article/10.3390/biomedicines12030534/s1, File S1: The dysregulated transcripts obtained from duplicate next-generation sequencing experiments comparing macrophages treated with CLP-exo and Control-exo; File S2: The dysregulated proteins that were changed by more than 2-fold in the duplicate iTRAQ proteomic analysis of the exosomal protein content of CLP-exo and Control-exo; File S3: Survival rate of the C57BL/6 mice receiving CLP of high-grade sepsis model (*n* = 20).

## Abbreviations

Acetyl-CoA	acetyl coenzyme A
CLP	cecum ligation and puncture
DLS	dynamic light scattering
iTRAQ	isobaric tags for relative or absolute quantitation
LncRNAs	long non-coding RNAs
LPS	lipopolysaccharide
Mbnl1	muscleblind-like protein 1
Mbnl2	muscleblind-like protein 2
MEM	multi experiment matrix
MIR17HG	miR-17-92a-1 cluster host gene
NGS	next-generation sequencing
PDI	polydispersity index
RAC2	ras-related C3 botulinum toxin substances 2
siRNA	small interfering RNA
STAT	signal transducer and activator of transcription
TEM	transmission electron microscopy
TLR	toll-like receptor
