# Supplementary figures and images for: Circulating Exosomes from Septic Mice Activate NF-κB/MIR17HG Pathway in Macrophages
Source: Biomedicines. 2024 Feb 27;12(3):534. doi: 10.3390/biomedicines12030534 (PMC10968321; doi:10.3390/biomedicines12030534)

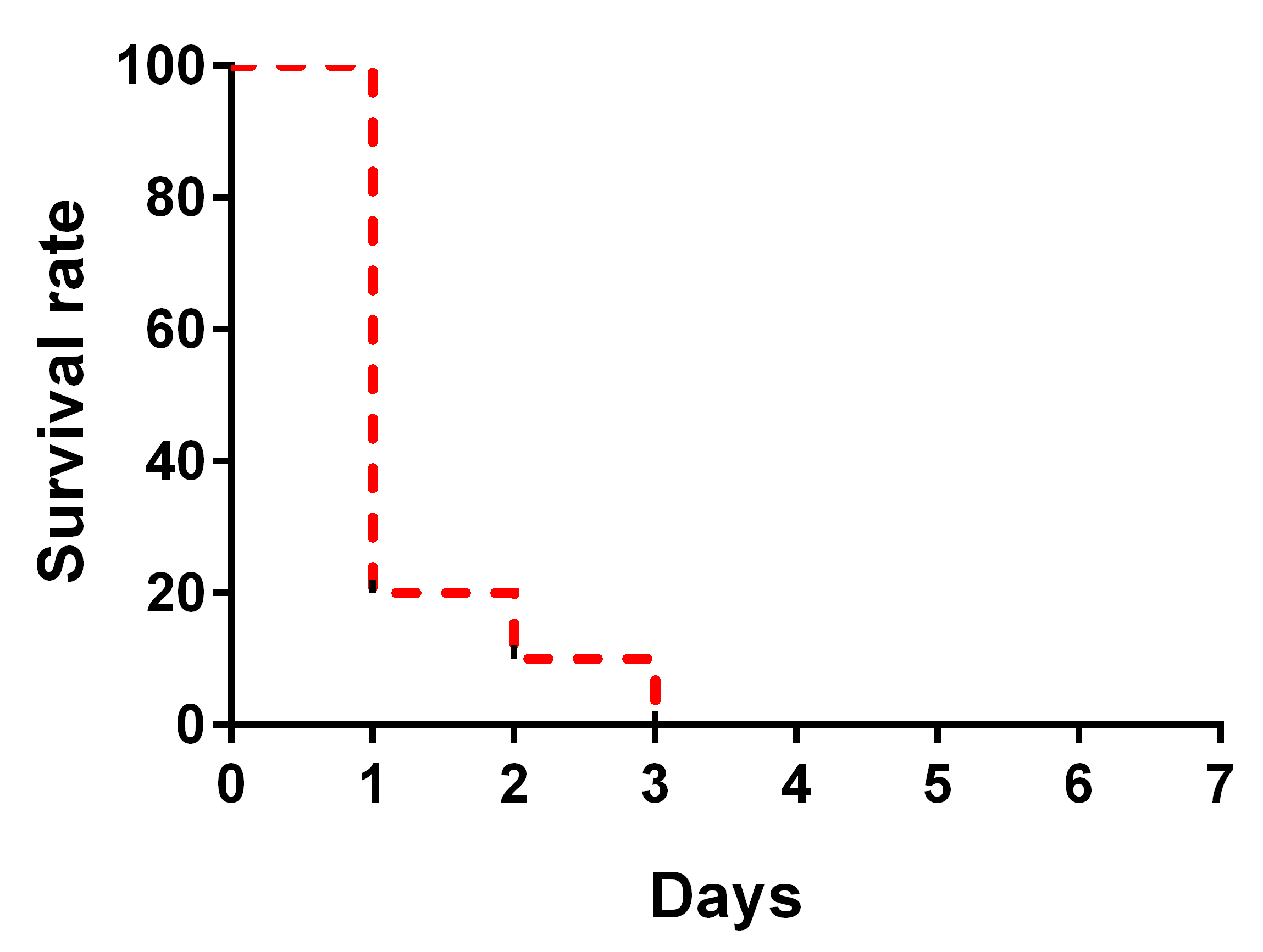

Supplement: Supplementary file 1 [file biomedicines-12-00534-s001.zip › File S3. Survival rate of the C57BL6 mice receiving CLP of high-grade sepsis model.tif]
